# The impact of policy on the intangible service efficiency of the primary health care institution- based on China’s health care reform policy in 2009

**DOI:** 10.1186/s12939-018-0901-2

**Published:** 2019-01-21

**Authors:** Yao Leng, Weiwei Liu, Nanzi Xiao, Yannan Li, Jing Deng

**Affiliations:** School of Public Health and Management, Chongqing Medical University, the Research Center for Medicine and Social Development, the Collaborative Innovation Center for Social Risk Governance in Health, No. 1 Medical School Road, Yuzhong District, Chongqing, 400016 China

**Keywords:** The impact, Intangible service efficiency, The primary health care institution, Health care reform

## Abstract

**Objectives:**

Analyzing the impact of the China’s health care reform policy in 2009 on the intangible service efficiency of PHCI and exploring the way to improve the service efficiency of PHCI.

**Methods:**

The Malmquist productivity index based on the Data Envelopment Analysis (DEA) was used to measure the variation of TFP and its decomposition of PHCI before and after the implementation of the health care reform policy in 2009. Then, the Tobit model was applied to estimate the key factors affecting the improvement of the intangible service efficiency of PHCI.

**Results:**

The number of health resources and the intangible service efficiency of PHCI have increased obviously since the implementation of the China’s health care reform. The growth of intangible service efficiency of PHCI was mainly affected by the technical progress, but the management level and scale efficiency change have not yet played an important role.

**Conclusions:**

The growth of intangible service efficiency in China’s PHCI still belongs to input growth rather than efficiency growth. In the future, the technical progress and improvement in the management level are the key measures to promote the intangible service efficiency of PHCI.

## Background

To achieve the goal of universal access to basic medical and health services, the Chinese government implements a health care reform policy in 2009. This reform incorporates five aspects: first, speeding up the construction of the fundamental medical security system; second, establishing the national basic drug system; third, improving the primary health care service system; fourth, promoting the equalization of basic public health services; fifth, facilitating the pilot reform of public hospitals. Those five aspects involved in the reform policy are to meet the need of residents’ basic medical and health services, which depends on the improvement of service efficiency and service quality of the Primary Health Care Institution (PHCI) [1–3]. Because PHCI is a crucial part of China’s health care system, it is the “gatekeepers” of Chinese residents’ health level. PHCI in China consists of community health service centers, community health service stations, township health centers and village clinics. It is provided with medical technical guidance and training staffs by county-level hospitals. PHCI offers basic public health services and medical services to residents of the agency’s service radiation areas.

The policy document of health care reform in 2009, the Chinese government proposes many reform measures for PHCI. For example, the central government plans to establish 29,000 township health centers in rural areas, and build 3700 community health service centers and 11,000 community health service stations in the city. The government also puts forward to cancelling the drug price addition in the medical institution and adjusting the residents’ medical insurance policy to promote urban and rural residents to gradually enjoy equalization of basic public services. At present, China’s health care reform policy is altering the way people receive health care services. For example, in 2017, the number of inpatients in PHCI accounted for 55.4% and that of outpatients accounted for 18.2% of the total. Those statistics show that PHCI plays an important role in residents’ health level, which is expected to meet the demand of residents’ public health and basic medical service. At the same time, it also reflects that the health care reform in 2009 has already had an impact on PHCI.

Many Chinese scholars measure the impact of the China’s health care reform in 2009 on PHCI, but their results are inconsistent. Some studies suggest that China’s health care reform in 2009 increases the health resources of PHCI, so they think the reform exerts great influence on PHCI [4, 5]. However, some studies manifest that reforms only augment the health resources of PHCI, but those health resources are not well utilized. Therefore, they believe that the reform has less impact on PHCI [6]. Most of them adopt qualitative methods rather than quantitative methods, and they merely measure changes in health resources of PHCI without combining health resources with service efficiency to assess the impact of the reform on PHCI. Therefore, the conclusions of those studies are open to question. Based on the aforementioned analysis, this paper would comprehensively analyze the impact of the China’s health care reform in 2009 on PHCI in combination with changes in health resources and service efficiency before and after the implementation of reform.

Scholars normally use the growth rate of Total Factor Productivity (TFP) to indicate changes in institutional service efficiency, for example, Sherman, etc. [7–9]. The growth rate of TFP indicates that while the inputs (including capital, labour and land) remain unchanged, the production volume still increases thanks to the purely technical change, which includes improvements in knowledge, economies of scale, and the organizational management level. So, this productivity growth brought by the purely technical change is called as intangible service efficiency in this article. Based on the research of Fare who used Data Envelopment Analysis (DEA) and the Malmquist production index to calculate the TFP, the scholar begins to introduce TFP into the efficiency study of the health service system, for example, Z. Lu-lu, etc. [10–14].

This article not merely intends to study the changes in the intangible services efficiency of PHCI, but also to study the key factors affecting the changes in service efficiency of PHCI. According to literature analysis, this article selects the Tobit model to analyze the variations of key factors influencing the intangible service efficiency of PHCI before and after reform. The classic Tobit model is a promotion of the probit regression proposed by the Nobel economist James Tobin in 1958 when he analyzed the expenditure of household durable goods [15]. Since then, many scholars have adopted it to analyze the influencing factors when the dependent variable is greater than 0 and continuous distribution, for instance, Xingbai Xu, Gong Qi etc. [16–18]. The dependent variable of this paper is the TFP value, which is significantly greater than 0, so the Tobit model is devoted to analyze the influencing factors of the invisible service efficiency of PHCI before and after reform.

Therefore, it is feasible and significant that this article uses the DEA-Malmquist production index to study the impact of China’s health care reform policy on the intangible service efficiency of PHCI, and then uses the Tobit model to measure the key factors enhancing the intangible service efficiency of PHCI. We believe that the study of the influence of the China’s health care reform policy in 2009 on PHCI is conductive to the Chinese government to develop better policies for the improvement of the service efficiency of PHCI. It is also beneficial to China’s primary health service providers to improve the weak links in the process of health services. And this study also provides a reference for other governments to develop correct health care reform policies.

## Methods and data

### Methods

TFP measured by the DEA-Malmquist productivity index is widely used in the evaluation of mechanism operation efficiency, for it is flexible when dealing with the multiple input and output and it can be objective when measuring the dynamic trend of the efficiency change of the medical institution. The earliest research on TFP was Abramovitz, Solow, etc., and they found that in addition to tangible production factors, which would increase production, the purely technical change would also increase production [19, 20]. They called it “Solo surplus”, which increased the production by intangible production factors. Later, “Solo surplus” developed into TFP. Many scholars have studied the measurement method of TFP, among whom the most representative is Fare [21–24]. In 1994, he proposed a non-parametric method based on the DEA-Malmquist productivity index and used it to measure the change of TFP.

Fare got the distance function form of the Malmquist productivity index via using the geometric means of two Malmquist productivity indexes to calculate productivity change based on Caves, who used one input and one output to measure the Malmquist productivity index [25]. The following is the form of the distance function of the Malmquist productivity index:

$$ {\displaystyle \begin{array}{l}{M}_0\left({x}^t,{y}^t,{x}^{t+1},{y}^{t+1}\right)=\frac{s_0^t\left({x}^t,{y}^t\right)}{s_0^t\left({x}^{t+1},{y}^{t+1}\right)}\times \frac{d_0^t\left({x}^{t+1},{y}^{t+1}/ VRS\right)}{d_0^t\left({x}^t,{y}^t/ VRS\right)}\sqrt{\frac{d_0^t\left({x}^{t+1},{y}^{t+1}\right)}{d_0^{t+1}\left({x}^{t+1},{y}^{t+1}\right)}\times \frac{d_0^t\left({x}^t,{y}^t\right)}{d_0^{t+1}\left({x}^t,{y}^t\right)}}\\ {}\kern8.2em = ECH\times TECHCH\\ {}\kern8.2em = PECH\times SECH\times TECHCH\end{array}} $$

The Malmquist productivity index falls into two parts: efficiency change (ECH) and technical change (TECHCH). The efficiency change can be decomposed into two parts: pure efficiency change (PECH), which also refers to the institutional management level, and scale efficiency change (SECH). In line with the research of Fare: the TFP value > 1 means that the organization productivity is improved. If PECH value > 1, the management level has improved. TECHCH> 1 signifies that the organization’s technology has improved. SECH > 1 means that the production efficiency has been improved with the increase of the input factor, and economies of scale have been realized. Conversely, if the above indicators < 1, the corresponding efficiency is deteriorating.

The Tobit model refers to a type of model, in which the dependent variable is continuously unilateral distribution and greater than 0 or equal to 0. It is also known as a censored regression model or a regression model of the limited dependent variable, which means that the observed value of the dependent variable is subject to certain restrictions. The obtained observation therefore does not fully reflect the actual state of the dependent variable. Since the dependent variables (TFP, TECHCH, PECH and SECH) in this article are continuously unilateral distribution, which far outweighs 0, a constrained variable model is required. It is of necessity to establish a restricted dependent variable model to analyze the changes of the main influencing factors of PHCI before and after the reform. And the Tobit model is a mature, simple, and reliable restricted dependent variable model. Therefore, we choose this model to analyze the influencing factors. To analyze whether the intangible service efficiency of PHCI has improved after China’s health care reform in 2009, this article uses a TFP value of 1 as a key node for building the Tobit model. Its’ basic form is as follows:

$$ {Z}_{it}=\left\{\begin{array}{cc}\mathrm{C}{x}_{\mathrm{i},t}+{\varepsilon}_{it},& {\mathrm{Cx}}_{i\mathrm{t}}+{\varepsilon}_{it}>1\\ {}1,& {\mathrm{Cx}}_{it}+{\varepsilon}_{it}\le 1\\ {}{\varepsilon}_{it}\sim N\left(0,{\sigma}^2\right)& \end{array}\right. $$

### Data description and data source

The DEA-Malmquist production index measures TFP after removing the increase of production from tangible production factors. Hence, it is necessary to choose indicators that represent tangible production factors when selecting input indicators. This article combines the characteristics of Chinese data with the requirements of the DEA-Malmquist production index to select three input indicators.

We select the quantity of doctors and nurses instead of the human resource input, and take the number of hospital beds to replace the medical equipment input (Fixed assets) referring to the practice in articles of Afonso, etc. [26, 27]. Since the output of health service of PHCI is difficult to quantify, the output indicators selected in this article are the amount of inpatients and outpatients.

A country’s macroeconomic development level will affect the income level of residents, the development of medical institutions and the state’s financial input to medical institutions. The infrastructure and the human resource input of the medical institution will directly impact the medical technology and the service level of medical establishments. The distribution of medical institutions in the region will also affect the residents’ medical treatment behavior and the choice of medical institutions. The education level of the residents themselves will influence the residents’ health care awareness. Therefore, this study selects the following six indicators to analyze the influencing factors. From the macro aspects, elements of the national economic development level, public health investment and the distribution of medical institutions are selected. From the micro aspects, the infrastructure input, the human resource input and the education level of residents were chosen. The relevant data of PHCI are from the China Health Statistics Yearbook in 2006~2015 and the China National Data Network.

In accordance with the decomposition value of TFP and the selection of influencing factors, the Tobit models are established:

$$ {\mathrm{TFP}}_{\mathrm{i},\mathrm{t}}={\mathrm{C}}_0+{{\mathrm{C}}_1}^{\ast }{\mathrm{GDP}}_{\mathrm{i}\mathrm{t}}+{{\mathrm{C}}_2}^{\ast }{\mathrm{INF}}_{\mathrm{i}\mathrm{t}}+{{\mathrm{C}}_3}^{\ast }{\mathrm{HR}}_{\mathrm{i}\mathrm{t}}+{\mathrm{C}}_4\cdot {\mathrm{EL}}_{\mathrm{i}\mathrm{t}}+{C_5}^{\ast }{ID}_{it}+{C_6}^{\ast }{PHI}_{it}+{\varepsilon}_{it} $$

$$ {\mathrm{TECHCH}}_{\mathrm{i},\mathrm{t}}={\mathrm{C}}_0+{{\mathrm{C}}_1}^{\ast }{\mathrm{GDP}}_{\mathrm{i}\mathrm{t}}+{{\mathrm{C}}_2}^{\ast }{\mathrm{INF}}_{\mathrm{i}\mathrm{t}}+{{\mathrm{C}}_3}^{\ast }{\mathrm{HR}}_{\mathrm{i}\mathrm{t}}+{\mathrm{C}}_4\cdot {\mathrm{EL}}_{\mathrm{i}\mathrm{t}}+{C_5}^{\ast }{ID}_{it}+{C_6}^{\ast }{PHI}_{it}+{\varepsilon}_{it} $$

$$ {\mathrm{PECH}}_{\mathrm{i},\mathrm{t}}={\mathrm{C}}_0+{{\mathrm{C}}_1}^{\ast }{\mathrm{GDP}}_{\mathrm{i}\mathrm{t}}+{{\mathrm{C}}_2}^{\ast }{\mathrm{INF}}_{\mathrm{i}\mathrm{t}}+{{\mathrm{C}}_3}^{\ast }{\mathrm{HR}}_{\mathrm{i}\mathrm{t}}+{\mathrm{C}}_4\cdot {\mathrm{EL}}_{\mathrm{i}\mathrm{t}}+{C_5}^{\ast }{ID}_{it}+{C_6}^{\ast }{PHI}_{it}+{\varepsilon}_{it} $$

$$ {\mathrm{SECH}}_{\mathrm{i},\mathrm{t}}={\mathrm{C}}_0+{{\mathrm{C}}_1}^{\ast }{\mathrm{GDP}}_{\mathrm{i}\mathrm{t}}+{{\mathrm{C}}_2}^{\ast }{\mathrm{INF}}_{\mathrm{i}\mathrm{t}}+{{\mathrm{C}}_3}^{\ast }{\mathrm{HR}}_{\mathrm{i}\mathrm{t}}+{\mathrm{C}}_4\cdot {\mathrm{EL}}_{\mathrm{i}\mathrm{t}}+{C_5}^{\ast }{ID}_{it}+{C_6}^{\ast }{PHI}_{it}+{\varepsilon}_{it} $$

Among them, TFP_i, t_, TECHCH_i, t_, PECH_i, t_ and SECH_i, t_ represent the total factor productivity and its decomposition value (technical change, the management level and scale efficiency change) in 31 provinces of China from 2006 to 2015, respectively. The meanings of GDP, INF, HR, EL, ID and PHI are shown in the Table 1. C_0_ is the constant term of the model. C_1_, C_2_, C_3_, C_4_ and C_5_ are the regression coefficients of each variable. When the value of TFP_i, t_, TECHCH_i, t_, PECH_i, t_ and SECH_i, t_ is greater than 1, the true measurement value is taken, otherwise the value is 1. And the C and σ of the Tobit model are estimated by the maximum likelihood method. This article uses the software DEAP2.1 to calculate the value of TFP and utilizes the software STATA13.0 for Tobit regression analysis.

**Table 1:** The meaning of independent variables

Independent variable	Meaning
GDP	The per capita Gross Domestic Product in China.
INF	The number of beds per 1000 people in China’s PHCI.
HR	The number of doctors and nurses per 1000 people in China’s PHCI.
EL	The level of education of Chinese residents.
ID	The number of primary care institutions per square kilometre.
PHI	The government public health investment.

## Results

### Health resources analysis of China’s primary health care institution

**Fig. 1:**
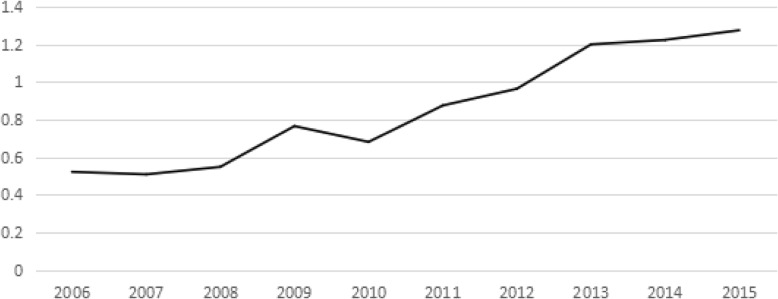
The number of medical staffs of PHCI during 2006~2015(Per 1000 people)

The number of medical staffs per 1000 people in China’s PHCI indicated an obvious upward trend in 2006~2015, rising from 0.52 in 2006 to 1.27 in 2015, and its growth rate was 144%. The quantity of medical personnel per 1000 people of China’s PHCI showed a growth peak in 2009, but it decreased significantly in 2009~2010. After 2010, it showed a steady growth tendency and the growth was not prominent after 2013. In 2009, China increased its financial investment in PHCI and used policy guidance to promote the growth of medical staffs in PHCI. (See Fig. 1).

**Fig. 2:**
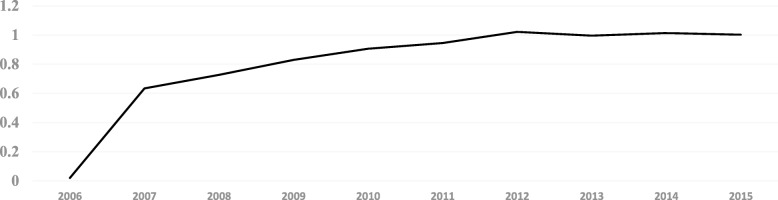
The number of hospital beds of the PHCI in China during 2006~2015(10 thousand)

The number of hospital beds per 1000 people of PHCI has been increased from 0.02 in 2006 to 1.002 in 2015. And its growth rate was close to 4910% with the average growth rate of 546% during the period of 2006~2015. The high growth rate of hospital beds of PHCI was mainly due to the growth of 2006 ~ 2007, and the change of the growth rate in 2013~2015 was not evident. (See Fig. 2).

**Fig. 3:**
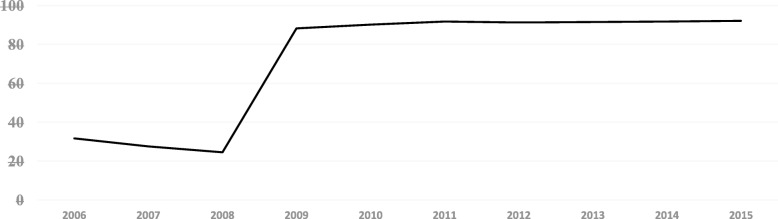
The number of the PHCI in China during 2006~2015(Per 1000 people)

The number of PHCI in China reflected a downward trend from 2006 to 2008, while the performance in 2008~2009 showed a large growth tendency. The number of PHCI increased slowly from 2009 to 2015. On the eve of the implementation of the health care reform policy in 2009, the Chinese government integrated and evaluated PHCI around the country, and “cleaned up” those PHCI that failed to meet the requirements. This brought about the reduction of the number of PHCI in 2006~2008. China’s document of health care reform in 2009 clearly stated that it would increase the number of PHCI. The definition of PHCI was redefined and the statistics on the number of PHCI in the China Health Statistics Yearbook followed this new definition. Therefore, the implementation of reform and the difference in the statistical caliber led to a high growth in the number of PHCI during 2008~2009. (See Fig. 3).

### The TFP analysis of the primary health care institution in China

The TFP value of PHCI during the period of 2009~2015 (after the implementation of the China’s Health Care Reform Policy in 2009) was greater than that during 2006~2009(before the implementation of the China’s Health Care Reform Policy in 2009). Among the 31 provinces in China, the TFP value of PHCI was greater than 1 expecting from the provinces of Fujian, Jiangxi and Guizhou. The TFP value of the four provinces of Beijing, Hebei, Zhejiang and Tibet were much bigger than other provinces, with the TFP value being 1.137, 1.122, 1.101 and 1.178, respectively. Among them, the increase in TFP of Tibet was the largest.

**Fig. 4:**
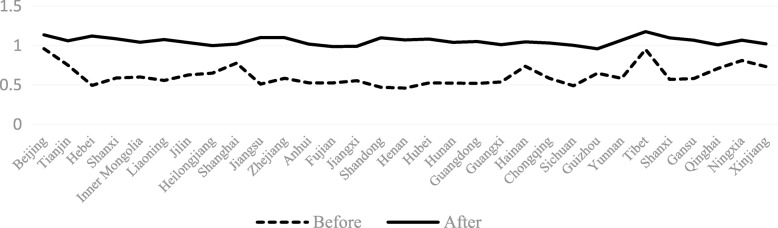
The change of TFP value in 31 provinces before and after the implementation of China’s health care reform policy

**Fig. 5:**
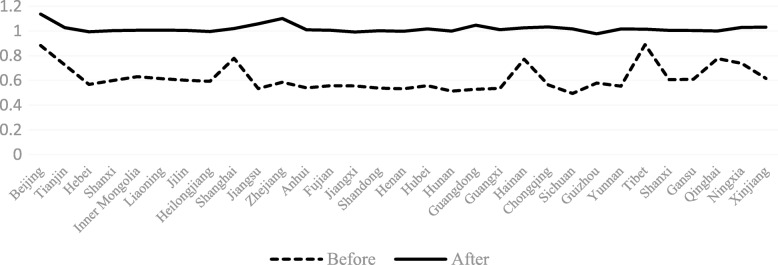
The change of TECHCH value in 31 provinces before and after the implementation of China’s health care reform policy

**Fig. 6:**
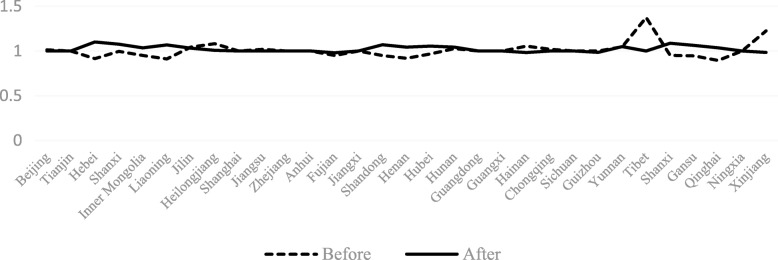
The change of PECH value in 31 provinces before and after the implementation of China’s health care reform policy

**Fig. 7:**
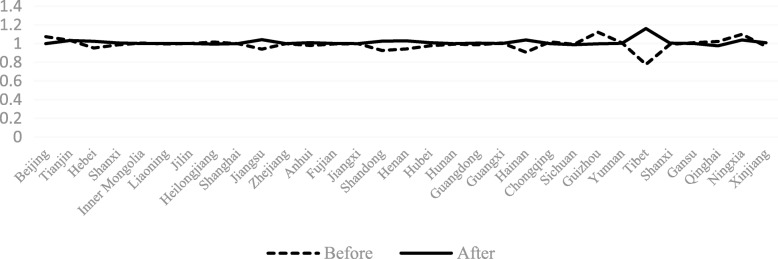
The change of SECH value in 31 provinces before and after the implementation of China’s health care reform policy

The TECHCH value of PHCI during 2009~2015 was significantly higher than that during 2006~2009. Apart from the provinces of Hebei, Heilongjiang, Jiangxi, Henan, Hunan and Guizhou, the TECHCH value was greater than 1 during 2009~2015. Among the 31 provinces in China, the TECHCH value of Beijing province was the largest, with the value of 1.14. The PECH and SECH value of PHCI didn’t increase during the period of 2006~2015 except a few provinces (such as the Hebei province and the Shandong province). Even in some provinces, such as Beijing province and Tibet province, the value of PECH and SECH was smaller than before. (See Figs. 4, 5, 6, 7).

**Fig. 8:**
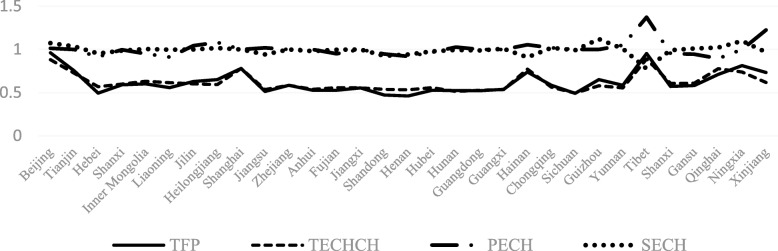
The change of TFP and its decomposition value in 31 provinces before the implementation of China’s health care reform policy

**Fig. 9:**
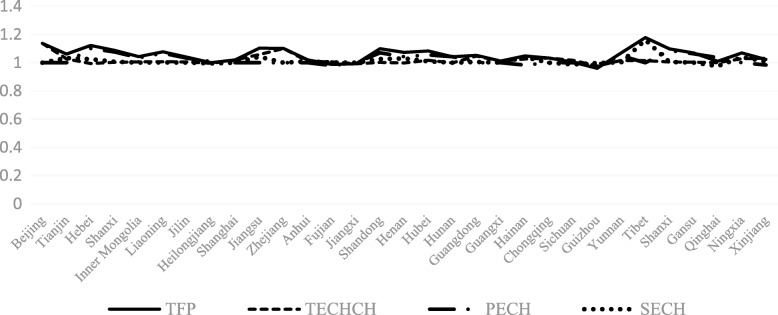
The change of TFP and its decomposition value in 31 provinces after the implementation of China’s health care reform policy

**Fig. 10:**
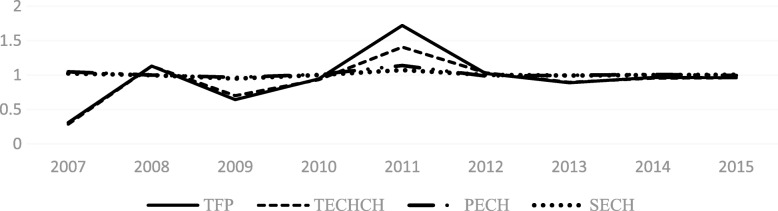
The value of TFP and its decomposition of PHCI in China during 2006~2015

From Figs 4, 5, 6, 7, 8 and 9 and Fig. 10, the following results could be obtained. First, after the health care reform, the values of TFP and TECHCH of PHCI in all provinces increased significantly, and they were significantly greater than 1. But the changes in PECH and SECH values were not apparent (Figs. 4, 5, 6 and 7). Second, before the implementation of health care reform, the values of PECH and SECH far outweighed TFP and TECHCH; after the health care reform, the values of TFP and TECHCH were greater than PECH and SECH (Figs. 8, and 9). Third, during the period from 2006 to 2011, the TFP and TECHCH values of PHCI showed an upward trend of “interval”. Among them, the TFP and TECHCH values of PHCI in 2011 is the maximum. However, the growth in TFP and TECHCH values was not obvious after 2012, and there was even a downward trend (Fig. 10). Fourth, from a comprehensive perspective, the variation trend of TECHCH and TFP was consistent, so it can be considered that the increase in TECHCH was the main reason for the increase in TFP. In other words, the improvement of the intangible service efficiency of China’s PHCI was largely due to technical change.

### Analysis of influence factors of the primary health care institution’s service efficiency in China

Through the analysis of the Tobit model of TFP value (See Table 2), we found that the significant factors influencing the growth of TFP value of PHCI were the economic development level, the infrastructure input, the human resource input and the government health input before the implementation of the health care reform policy in 2009. The influence coefficients were 0.155, 0.811, − 1.24, and 15.721, respectively. Among them, the biggest influence factor on the growth of TFP value was the government health investment. In above four significant influence factors, the economic development level, the infrastructure input and the government public health input showed the positive effect on the growth of TFP value. The government health input reflected the biggest positive effect on the growth of TFP value, while the the human resource input was negative. After the implementation of reform policy, we found that only the human resource input had a significant influence on the growth of TFP value of PHCI at the 1% level, with the influence coefficient of − 0.814. This indicated that the human resource input had a negative effect on the growth of TFP value of PHCI in China, while other factors have no significant influence on TFP value.

**Table 2:** Results of Tobit model of TFP, TECHCH, PECH and SECH

Model para	TFP		TECHCH		PECH		SECH	
	Before	After	Before	After	Before	After	Before	After
Cons	0.750* (2.74)	1.637* (4.75)	0.806* (5.46)	1.370 (9.33)	0.79* (2.67)	1.205* (6.56)	1.059* (8.57)	0.976*(8.65)
GDP	0.155** (2.27)	0.017 (0.33)	0.070** (1.91)	0.007 (0.34)	0.043 (0.53)	−0.045 (−1.51)	− 0.032 (−0.97)	0.008 (0.49)
INF	0.811* (2.42)	−0.150 (−0.54)	0.242 (1.37)	−0.126 (−1.06)	0.59 (1.46)	−0.055 (−0.37)	− 0.248 (−1.53)	0.149 (1.54)
HR	−1.240* (−2.90)	−0.814* (−2.85)	−0.288 (−1.33)	− 0.334* (− 2.77)	−0.781*** (− 1.69)	0.189 (1.09)	0.105 (0.60)	−0.253* (− 2.46)
EL	−6.752 (−1.4)	1.964 (0.79)	0.329 (0.12)	1.157 (1.07)	−6.924 (−1.34)	0.057 (0.04)	0.772 (0.33)	−0.685 (−0.87)
ID	−0.821 (−1.42)	0.193 (0.80)	−0.434 (−1.41)	0.120 (1.18)	−0.773 (− 1.07)	−0.218 (− 1.49)	− 0.260 (−1.07)	− 0.056 (−0.69)
PHI	15.721** (1.81)	−5.832 (−0.99)	−1.910 (− 0.37)	−4.008 (−1.56)	17.301*** (1.75)	−5.24 (−1.59)	0.572 (0.13)	1.585 (0.88)
LR chi2(6)	16.58	19.58	8.36	26.75	10.19	20.13	6.81	11.70
Prob>chi2	0.0109	0.0033	0.21	0.0002	0.117	0.002	0.339	0.069
Sigma	0.361	0.702	0.207	0 .294	0.39	0.31	0.19	0.22
Loglikelihood	−39.582	−139.478	−25.276	−70.801	−39.043	−63.840	−20.936	−49.365

Note: When we do regression analysis, we do a logarithmic processing of the dependent variable

After analyzing the Tobit model of TECHCH value (See Table 2), we discovered that before the implementation of the China’s health reform policy in 2009, the economic development level showed the positive influence on the growth of TECHCH values of PHCI at the 5% level of statistical significance, with the influence coefficient being 0.07. After t he reform policy, the economic development level was no longer a key factor for influencing the growth of TECHCH value. However, the influence of the human resource input on the growth of TECHCH value was crucial, which was significant at the 1% level. And the influence coefficient was − 0.334. This means that the human resource input had a negative effect on the growth of TECHCH value of PHCI after the implementation of the health reform.

Through analyzing the Tobit model of PECH value (See Table 2), we found that before the implementation of China’s health reform policy in 2009, the human resource input and the government health input had great influence on the growth of PECH value of PHCI at the 10% level. The influence coefficients were − 0.781 and 17.301, respectively. This indicated that the impact of the human resource input on the growth of PECH value of PHCI was negative, and the government health input was positive for the growth of PECH value. After the implementation of the reform in 2009, the impact of the influence factors in this study on the growth of PECH value of PHCI was not statistically significant.

After analyzing of the Tobit model of SECH value (See Table 2), we discovered that before the implementation of China’s health reform policy in 2009, the impact of the influence factors selected in this study was not statistically significant on the growth of SECH value. And after the implementation of the reform, the human resource input had the significant influence on the growth of SECH value at the 1% level, with the influence coefficient of − 0.253. It showed that the health human resource input of PHCI in China had the adverse impact on the growth of SECH value.

## Discussion

The health resources of PHCI in China increased rapidly after the implementation of health care reform in 2009,but this rapid growth benefited from the reform policy was not sustainable. In the implementation of the reform policy in 2009, the Chinese government proposed the target of improving the primary health care system from the perspective of increasing the number of PHCI, medical equipment and medical staffs. In this article, the health resources in China’s PHCI have witnessed a phenomenon of the “sharp increase”. For example, the number of the medical staffs increased by 40% and the quantity of PHCI increased by 200% in 2008~2009. Those data showed that the rapid growth of health resources in PHCI during 2008~2009 was dominated by the government’s reform policy. This conclusion is consistent with other scholars [28–31]. China’s economic development has entered a relatively stable period, and China is a country with large regional economic differences and the industrial development distinction. Thus, the Chinese government need to coordinate the development of industries and regions through the financial input. If the long-term increase in financial investment to PHCI will lead to a reduction in the financial input in other areas, it would directly affect the coordinated development of various fields. Therefore, the government’s financial input changed with the policy. The Chinese government adjusted the focus of health reforms in 2012, and the growth of health resources in PHCI slowed down. So, the rapid growth of health resources brought by the reform policy was not sustainable, and it would diminish as the focus of reform changes.

We find that the implementation of the China’s health care reform policy in 2009 has a significantly stimulative influence to the growth of the intangible service efficiency of PHCI, but this increase still belongs to input growth instead of efficiency growth. Although we have measured the growth of intangible service efficiency of PHCI after removing the influence of tangible input factors in this article, we observe that the existence of certain tangible production factors will promote the technical progress of PHCI. The implementation of China’s health care reform policy in 2009 has resulted in a significant increase in the number of medical staffs and advanced medical equipment of PHCI, and this has led to advances in the intangible service efficiency of PHCI. While implementing the health care reform, the Chinese government has re-established the system of “the first diagnosis at the PHCI” and strengthened it continuously. The Chinese government has also adjusted the medical insurance policy to encourage and guide residents to go to the PHCI to see a doctor when they are sick. Those measures provide a sufficient “market” for PHCI. Consequently, the intangible service efficiency of PHCI has been improved.

However, this improvement of intangible service efficiency is not sustainable, for the growth of PHCI still pertains to input growth other than efficiency growth. First, technical change fails to drive the intangible service efficiency of PHCI for a long time. Chen found that the management efficiency and scale efficiency change only affected the TFP growth of PHCI within a certain range, but technical progress was the key factor [32]. The improvement of the intangible service efficiency of China’s PHCI was mainly driven by the technical progress, but the technical progress of PHCI was not obvious after 2012. After 2012, the focus of the China’s health care reform was transferred to general hospitals, which brought about a reduction in government spending on production factors in PHCI. At that time, the technical progress of PHCI was not significant, meaning that the medical technology of PHCI was not fundamentally improved and the technical progress of PHCI was mainly caused by the increase of the production factor input. Second, the low management ability and scale efficiency also restricted the growth of intangible service efficiency of PHCI into efficient growth. The management level and scale efficiency change were also the part of the intangible service efficiency of PHCI. But the management level and scale efficiency of PHCI have not been improved after the implementation of the reform policy in 2009, which is consistent with the study of other scholars [33]. Those reasons explain that the growth of intangible service efficiency of PHCI still belongs to input growth rather than efficiency growth.

The impact of the economic development level on the growth of intangible service efficiency of PHCI is not significant after the health care reform. The direct impact of the economic development level on PHCI is mainly reflected in the following aspects. The level of regional economic development affects the local government’s investment in health resources and the salary level and willingness of medical staffs to work. Since the PHCI of China is established by the government with public welfare and mainly provides basic public health services for residents, the government’s financial input plays an important role in PHCI. Therefore, the China’s health reform adjusts the financial input to coordinate differences mainly in the allocation of health resources due to regional economic development. Through raising the salary level of medical staffs of PHCI and expanding their social welfare, the government guides and supports the general practitioners to work in PHCI. The implementation of the health care reform policy has not merely narrowed the gap in the allocation of health resources between regions, but also effectively increased the intangible service efficiency of PHCI owing to the guiding role of government policies to a large extent. Those changes brought by the reform policy have made the impact of the economic development level on improving the intangible service efficiency of PHCI no longer significant. The influence of the economic development level between regions on PHCI has become smaller after the reform in the Tobit model, which also confirms this conclusion.

After the implementation of the health care reform policy, the impact of the infrastructure input on the growth of intangible service efficiency of PHCI is not significant any more, and it has changed from positive to negative. Before the implementation of the reform policy, the PHCI lacks relevant infrastructure, such as advanced medical equipment, but the implementation of reform policy has improved this situation. For example, the implementation of the reform policy has offered many advanced medical examination equipment to PHCI. But the medical technicians at PHCI are not fully trained, which has led to the situation where no one is able to use advanced medical equipment in PHCI. A mass of medical equipment in PHCI is in an idle state after the implementation of the reform policy, which means that the increase in inputs and outputs is not matched [34]. Therefore, the infrastructure input has failed to promote the intangible service efficiency of PHCI. At present, this negative impact is not significant. If the government or PHCI do not take measures, this negative impact will become more and more apparent, and finally become a key factor hindering the growth of intangible service efficiency of PHCI.

After the implementation of China’s health care reform policy, the human resources of PHCI have an adverse impact on the improvement of the intangible service efficiency of PHCI, and this negative impact is getting smaller. First of all, the number, quality, structure and distribution of medical staffs in PHCI directly affect its service function and determine its service level [35–37]. The implementation of reform policy has improved the situation of serious shortage of medical staffs in PHCI, but there are still issues such as unreasonable medical staffs structure, low academic qualifications and small development space [38]. Therefore, the health care reform policy in 2009 could only reduce the negative impact of human resources on the improvement of the intangible service efficiency of PHCI, but it could not turn negative influence into positive influence. Secondly, the performance pay of medical staffs of PHCI has problems such as low basic salary and the small proportion of reward performance, which directly lead to the low enthusiasm and the loss of medical staff in PHCI [39, 40]. Therefore, the human resources restrict the improvement of the intangible service efficiency of PHCI.

After the implementation of health care reform policy, government public health investment has unfavorable effect on improving the intangible service efficiency of PHCI, but it is not statistically significant. The government’s public health investment aims to control the huge difference of basic health care caused by the distinction in the economic development level among different regions, and it plays a role of the “baton”. In 2009, China’s health care reform has increased investment in PHCI in order to develop basic health care. In an ideal situation, it should promote the intangible service efficiency of PHCI. For example, public health investment has a positive impact on the enhancement of the scale efficiency of PHCI (See Table 2). However, if the government’s public health investment is not entirely and rationally utilized, the increase in government public health investment would lead to the resource redundancy. This will generate a mismatch between the input and the output of PHCI, thus it will hinder the intangible service efficiency of PHCI. Whether the government’s public health investment is properly used is closely related to the management staffs of relevant government departments and PHCI, which is consistent with the above conclusion that the capacity of management staffs of PHCI is insufficient.

The educational level and institutional density have little effect on the service efficiency of PHCI before and after the health care reform in 2009, and they are not discussed here.

## Conclusions and suggestions

Through the aforementioned analysis, we draw the following conclusions: First of all, after the implementation of the health care reform in 2009, the intangible service efficiency of China’s PHCI increased significantly, but this growth is not sustainable. The reasons are that the growth of intangible service efficiency in China’s PHCI is due to the increasing input factors of government and the growth of intangible service efficiency is still pertains to input growth other than efficiency growth. Secondly, the technical progress of PHCI is the key factors that restrict the growth of intangible services efficiency in China’s primary health care institutions, and the management level of PHCI and the full utilization of health resources are also important factors. According to our main conclusions, we find that there are four key measures to improve the intangible service efficiency of PHCI after China’s 2009 health care policy reform.

First, we should promote the advancement and innovation of medical technology in PHCI. At present, the medical technology of medical staffs is mainly based on conventional diagnosis, treatment and nursing, lacking compound talents with professional medical knowledge. The knowledge includes preventive health care nutrition rehabilitation, psychological intervention and electronic medical records construction. The deficiency of medical staffs on medical technology would limit the development of the intangible service efficiency of PHCI. In the article, there are two mainly feasible measures to eliminate this deficiency:making full use of the education resources in the medical college and training the original medical staffs of PHCI. It will make them the new type of compound talents with the medical technology of preventive care, nutritional rehabilitation and psychological intervention, etc. The PHCI can introduce the new type talents of medical technology directly from the society in order to build a suitable health care team.

Second, we should improve the management ability of managers of PHCI. The characteristics of PHCI determine that their operation has certain peculiarities. The managers of PHCI not only manage the daily affairs of the institutions, but also have certain control over the technical level to prevent medical accidents. Therefore, in the daily work, it is necessary to pay more attention to the hospital management work. The PHCI should have two management systems: the one is the management system responsible for medical technology and medical services, which requires medical technicians with advanced medical technology; the other is the coordination over the resources of persons, finance and materials, which requires people to have professional management knowledge and the economic background. Therefore, we can improve the management level of managers in PHCI from two aspects. First, training the existing medical technicians with professional management knowledge to make them become the management talents with management thinking and advanced medical technology. Second, we are to introduce managerial staffs with the medical background to PHCI.

Third, we ought to make reasonable use of existing health resources to avoid the waste of resources and the redundancy. After the health care reform in 2009, the health resources of PHCI have the serious redundancy and China has a large population with limited health resources. Therefore, China’s PHCI cannot blindly ask the government to increase the input of health resources and the financial input. Instead, they should make the best of extent health resources to improve the service efficiency of PHCI. PHCI can send health care workers to higher-level hospitals to learn how to use related advanced medical devices. Higher-level medical institutions can also send personnel from inspection departments to PHCI for training purposes. PHCI can also directly introduce graduates of laboratory science and diagnostics from medical colleges. Those measures require the government to have corresponding support policies. For example, the government should give medical staff participating in training and the study a certain amount of money subsidies, support for professional title evaluation, etc. The government also should provide social welfare benefits such as preferential housing purchases, or directly increase their salary levels for newly introduced graduates from the medical college, etc.

Fourth, we should take a work mode that focuses on both disease prevention and chronic disease management. The purpose of PHCI is to ensure that Chinese residents enjoy equal basic medical care and reduce the occurrence and development of diseases. Instead of treating diseases, the work center of PHCI should be disease prevention, health education publicity, chronic disease management, etc. And the social benefits brought by disease prevention are far greater than the treatment of diseases. Therefore, improving the intangible service efficiency of PHCI should not only rely on the improvement of the efficiency of medical services, but also depend on disease prevention and chronic disease management. Hence, the PHCI should make efforts to prevent disease while providing basic medical services. The PHCI can enhance the awareness of disease prevention by strengthening health education publicity to reduce the incidence of diseases. For example, for chronic diseases, institutions can increase publicity from the aspects of the diet and health activities, guide residents to establish a healthy lifestyle, reduce the occurrence of chronic diseases and control their development.

## Limitations

First, for the service efficiency of medical institutions, the improvement of intangible service efficiency by purely technological efficiency change is a very important component. However, the improvement of service efficiency by tangible production factors is also an indispensable part. This article measures the impact of purely technological efficiency variation on service efficiency of primary health care institutions after excluding the impact of tangible production factors. Therefore, it has certain limitations on the overall service efficiency of primary health care institutions.

Second, as the quantitative indicators of the health input and the output in China’s primary health care institutions are not clear, this paper refers to other scholars’ practices to select input and output indicators. This may lead to some deviations of the research results, and it also results in the weakness of representation.

Third, the Tobit model in this article is directly selected for the analysis of influencing factors. We don’t take the multi-model comparative analysis from the research methods of other scholars, which will reduce the credibility of the research results. Nevertheless, the results of this study are still representative and authentic, and it can illustrate the impact of the implementation of China’s health care reform policy in 2009 on the intangible service efficiency of primary health care institutions.

## Abbreviations

DEA: Data Envelopment Analysis
ECH: The efficiency change
EL: Education level
GDP: Per capita Gross Domestic Product
HR: Human resources
ID: Institutional density
INF: Infrastructure level
PECH: The pure efficiency change
PHCI: The Primary Health Care Institution
PHI: Public health investment
SECH: The scale efficiency change
TECHCH: The technical change
TFP: Total Factor Productivity
